# Genes and Pathways Affecting Sheep Productivity Traits: Genetic Parameters, Genome-Wide Association Mapping, and Pathway Enrichment Analysis

**DOI:** 10.3389/fgene.2021.710613

**Published:** 2021-07-28

**Authors:** Seyed Mehdi Esmaeili-Fard, Mohsen Gholizadeh, Seyed Hasan Hafezian, Rostam Abdollahi-Arpanahi

**Affiliations:** ^1^Department of Animal Science and Fisheries, Sari Agricultural Sciences and Natural Resources University (SANRU), Sari, Iran; ^2^Department of Animal and Dairy Science, University of Georgia, Athens, GA, United States

**Keywords:** maternal genes, maternal pathways, GWAS, gene-set analysis, ewe productivity

## Abstract

Ewe productivity is a composite and maternal trait that is considered the most important economic trait in sheep meat production. The objective of this study was the application of alternative genome-wide association study (GWAS) approaches followed by gene set enrichment analysis (GSEA) on the ewes’ genome to identify genes affecting pregnancy outcomes and lamb growth after parturition in Iranian Baluchi sheep. Three maternal composite traits at birth and weaning were considered. The traits were progeny birth weight, litter mean weight at birth, total litter weight at birth, progeny weaning weight, litter mean weight at weaning, and total litter weight at weaning. GWASs were performed on original phenotypes as well as on estimated breeding values. The significant SNPs associated with composite traits at birth were located within or near genes *RDX*, *FDX1*, *ARHGAP20*, *ZC3H12C*, *THBS1*, and *EPG5*. Identified genes and pathways have functions related to pregnancy, such as autophagy in the placenta, progesterone production by the placenta, placental formation, calcium ion transport, and maternal immune response. For composite traits at weaning, genes (*NR2C1*, *VEZT*, *HSD17B4*, *RSU1*, *CUBN*, *VIM*, *PRLR*, and *FTH1*) and pathways affecting feed intake and food conservation, development of mammary glands cytoskeleton structure, and production of milk components like fatty acids, proteins, and vitamin B-12, were identified. The results show that calcium ion transport during pregnancy and feeding lambs by milk after parturition can have the greatest impact on weight gain as compared to other effects of maternal origin.

## Introduction

In sheep breeding, ewe productivity is the most important trait affecting profitability, and genetic progress in this complex trait can lead to more efficient lamb production (Hanford et al., 2003). In some countries such as Iran, where meat is the main sheep product, the productivity of a ewe flock usually has the greatest influence on profitability (Wang and Dickerson, 1991). An increase in sheep meat production could be achieved by increasing the number and weight of lambs weaned per ewe within a specific year (Duguma et al., 2002). Ewe productivity is normally defined as the total litter weight at weaning per ewe. It is one of the most common composite traits affected by many cooperative components linked to reproduction and growth, including age at puberty, ovulation, pregnancy, parturition, lactation, mothering ability, and lamb survival and growth (Snowder and Fogarty, 2009).

Ewe productivity is often regarded as an overall measure of lamb production capacity by ewes (Bromley et al., 2001). Composite traits are a combination of growth and reproductive traits. Therefore, genetic improvement of ewe productivity is a key target in sheep breeding programs (Duguma et al., 2002). Common composite reproductive traits in sheep are total litter weight at birth and total litter weight at weaning. These parameters can be used as good coordinates for the market, where producers are paid per kilogram of sheep and not per head (Abdoli et al., 2019). Although estimates of genetic parameters have already been reported for composite traits of different Iranian sheep breeds (Abbasi et al., 2012; Abdoli et al., 2019), there are limited reports on the genes and pathways that affect these traits. To our knowledge, there is only one published report about genes and genomic regions associated with composite traits in sheep (Abdoli et al., 2019).

Due to a strict threshold used in GWASs to find significant SNPs, several poorly associated SNPs are always ignored. An alternative strategy is to add gene set analysis as a complement approach after GWAS (Dadousis et al., 2017). In this approach, a set of genes with some common functional features (e.g., being a member of a specific pathway) are identified by significant SNPs of GWAS, although with a less stringent threshold. Then, these genes are tested for over-representation in a specific pathway (Wang et al., 2011). Relevant to this context, there has been a growing interest in gene set enrichment analysis (GSEA) in dairy cattle (Han and Peñagaricano, 2016; Dadousis et al., 2017; Neupane et al., 2018).

The Baluchi sheep is the largest sheep breed in number in Iran and is well-adapted to a wide range of arid subtropical environments from the northeast to the southeast of the country (Moradband et al., 2011). Due to very limited reports on genes and pathways affecting composite traits in sheep, the objective of this study was to use GWAS and GSEA to unravel the genomic architecture underlying ewe productivity in Iranian Baluchi sheep.

## Materials and Methods

### Phenotypic and Genotypic Data

The data set consisted of 1,509 ewes with 3,916 and 3,635 records of birth weight and weaning weight (sheep at 90 days of age), respectively. Progeny birth weight (PBW), litter mean weight at birth (LMWB), and total litter weight at birth (TLWB) were used as maternal composite traits at birth. Also, progeny weaning weight (PWW), litter mean weight at weaning (LMWW), and total litter weight at weaning (TLWW) were considered maternal composite traits at weaning. The PWW trait was adjusted for birth weight according to the formula:

$$\mathrm{PWW}=(\mathrm{unadjusted}\mathrm{PWW}-\mathrm{PBW}/\mathrm{lamb}\mathrm{age}\left(\mathrm{day}\right)\mathrm{at}\mathrm{weaning}){}^{*}90,$$

and then used for TLWW and LMWW calculation. The LMWB and LMWW are arithmetic mean values of TLWB and TLWW traits. They were calculated for each lambing per ewe. Phenotypic correlation between traits ranged from 0.005 to 0.16. Correlation between the EBVs of traits ranged from 0.13 to 0.99. Correlation table of traits provided in Supplementary Table 7. The pedigree file included 4,727 animals with 178 sires, 1,509 dams, and 818 founders. Data were collected from 2004 to 2012 (9-year span) at Abbas Abad Baluchi sheep breeding station, located in Sarakhs city, Khorasan Razavi province, Iran. Descriptive statistics of the studied traits are presented in Table 1. The average litter size for all ewes and genotyped ewes were 1.45 and 1.56 lamb, respectively.

**TABLE 1:** Descriptive statistics of the studied traits for genotyped ewes.

Trait	N	Avg	SD	Min	Max	CV	Total N
PBW	436	4.26	0.72	2.30	6.80	0.17	3,916
TLWB	317	5.90	1.77	2.80	13.00	0.30	3,063
LMWB	317	4.40	0.75	2.70	6.80	0.17	3,063
PWW	398	20.31	4.06	9.10	34.60	0.20	3,635
TLWW	294	27.52	8.25	9.90	57.80	0.30	2,869
LMWW	294	20.97	3.98	9.90	34.60	0.19	2,869

**PBW, Progeny Birth Weight; TLWB, Total Litter Weight at Birth; LMWB, Litter Mean Weight at Birth; PWW, Progeny Weaning Weight; TLWW, Total Litter Weight at Weaning; LMWW, Litter Mean Weight at Weaning; N, Number of records; Avg, Average; SD, Standard deviation; Min, Minimum value; Max, Maximum value; CV, Coefficient of variation; Total N, Total number of observations used for EBVs calculation.**

Genotype data of 54,241 SNP markers were provided for 91 Baluchi ewes by the animal genetics group of Sari Agriculture Science and Natural Resource University (SANRU), Iran (Gholizadeh et al., 2014). Details of the feeding and management of Baluchi sheep were reported by Gholizadeh and Ghafouri-Kesbi (2015). In sampling the animals for genotyping, two criteria were considered: the selection of unrelated animals based on pedigree information and sampling those that represented the diversity of the breed. Missing markers were imputed using Beagle 5.2 (Browning et al., 2018) on a per chromosome basis. An effective population size equal to 134 was selected based on Tahmoorespur and Sheikhloo (2011). Also, a window size of 1 Mb with an overlap of 200 kb were set. The GenABEL package (Aulchenko et al., 2007) was used for quality control in R software (R Core Team, 2021). Genotyping call rate less than 95% was applied to filter out individuals. Furthermore, SNPs with unknown genomic location, those that were monomorphic or had minor allele frequency less than 0.01, those with genotype call rates less than 93%, and SNPs that departed from the Hardy–Weinberg equilibrium (for a *P*-value cut-off of 1 × 10^–6^) removed from the dataset and 45,342 SNPs were kept for the analysis.

### Genome-Wide Association Study

Here, we regressed progenies’ weights on mothers’ genotypes. As ewes had several lambing records, we used a repeatability model framework for the association analyses and could consider year and ewes age effects. We also could incorporate multiple records for each ewe in the analyses. Due to the small sample size, we used two different GWAS approaches to understand whether they confirm each other or not. In the first approach, phenotypes were used as the response variable, and in the second approach, EBVs were used as the response variable.

### Genome-Wide Association Mapping Using Phenotypes (pGWAS)

For the GWAS using phenotypes, the repeatability model was extended as follows:

$$y=X\,b+X_{S\,N\,P}\,\beta_{S\,N\,P}+Z\,u+W\,p\,e+e$$

where *y* is a vector of observations (ewe’s progenies weight); *b* is a vector of fixed effects, including the lamb’s sex, birth type, birth year, and the dam’s age at lambing; *u* is the vector of random direct additive genetic effects, *pe* is the vector of permanent environmental effects, and *e* is the vector of random residual effects. The lamb’s sex fixed effect was classified for all possible combinations and all traits except the PBW and PWW. The X, Z, and W are design matrices that relate individuals’ records to their fixed effects (*b*), additive genetic effects (*u*), and permanent environmental effects (*pe*), respectively. X_SNP_ is the incidence matrix for the SNP markers and *β*_SNP_ is the regression coefficient. In this case, the random effects have multivariate Gaussian (co)variance:

(up⁢ee|σu2,σp⁢e2,σe2)∼N⁢[0,(G⁢σu2000In⁢σpe2000IN⁢σe2)]

where G is the genomic relationship matrix, I is an identity matrix, *n* is the number of genotyped individuals with reproductive records (*n* = 91) and *N* is the total number of observations for the genotyped individuals (*N* = 294–436, depends on the trait). We can write the extended repeatability model as follows:

$$y=X\,b+X_{S\,N\,P}\,\beta_{S\,N\,P}+e$$

This model is the same as the model above if,

$$e∼N\,\left(0,V\right)\,w\,h\,e\,r\,e\,V=Z\,G\,Z^{{}^{\prime }}\,\sigma_{u}^{2}+W\,W^{{}^{\prime }}\,\sigma_{p\,e}^{2}+I_{N}\,\sigma_{e}^{2}.$$

In this approach, the *P*-value for SNP effects that occur in the original model can be calculated from the ratio of the *β*_*SNP*_ to its standard error (Wald test). An alternative approach is to use the following score test statistics that can be more computationally efficient, be asymptotically a normal standard, and one that approximates the Wald test,

$$Z=\frac{X_{S\,N\,P}^{{}^{\prime }}\,V_{\circ }^{-1}\,\left(y-X\,\hat{\beta }\right)}{\sqrt{X_{S\,N\,P}^{{}^{\prime }}\,V_{\circ }^{-1}}\,X_{S\,N\,P}}$$

but here *V*_∘_ is computed in the same way as *V* using a model where the SNP effects (*X_*SNP*_β_*SNP*_*) have been excluded and where $\hat{\beta }$ is computed from the model *y*=*Xb*+*X*_*SNP*_β_*SNP*_+*e*, assuming $e∼N\,\left(0,V_{\circ }\,\sigma_{e}^{2}\right)$. The analyses were performed using the R package RepeatABEL (Rönnegård et al., 2016).

### Genome-Wide Association Mapping Using Estimated Breeding Values (eGWAS)

The small number of available genotypes in this study can contribute to the low power of the association analysis, but the use of EBVs can increase the power to some extent as we have a better estimate of the actual genetic variance. EBVs can largely compensate for the limited number of genotypes to get reasonable estimates (Abdoli et al., 2018, 2019; Esmaeili-Fard et al., 2021). In this approach, first, we ran a pedigree-BLUP analysis using the classical repeatability animal model in the BLUPF90 software (Masuda, 2019), and breeding values of all animals (genotyped and not genotyped animals) were estimated for all traits. The lamb’s sex, birth type, birth year, and dam’s age at lambing were included in the model as fixed effects. Animal direct additive genetic and ewe permanent environmental effects were used as random effects. Variance components were estimated using the Restricted Maximum Likelihood (REML) approach, implemented in the AIREMLF90 software (Masuda, 2019). Accuracy (r) of EBVs were estimated as Henderson (1975) and Hayes et al. (2009):

$$r_{i}=\sqrt{1-S\,E_{i}^{2}}/\sigma_{a}^{2}$$

where SE_*i*_ is the standard error of EBV_*i*_, derived from the diagonal element of the inverted left-hand side in the mixed model equations (Henderson, 1975) and $\sigma_{a}^{2}$ is the additive genetic variance. Then we weight EBVs using the following formula:

$$W.E\,B\,V_{i}=\frac{1}{1-r_{i}^{2}}*E\,B\,V_{i}$$

Finally, weighted EBVs of genotyped animals were used as the response variable and SNP genotypes were fitted as the fixed effects in a GLM model as follows,

$$W.E\,B\,V\,s=X_{S\,N\,P}\,\beta_{S\,N\,P}+e$$

where *X*_*SNP*_ is the design matrix that relates weighted EBVs to SNP genotypes and *β*_*SNP*_ is the regression coefficient. The GenABEL (Aulchenko et al., 2007) package in the R environment was used for this analysis. Due to the use of the genomic and pedigree-based relationship matrix in GWA analysis, *p*-values were almost non-inflated (1.01 ≤ λ ≤ 1.07) for all traits, however, partial inflation was corrected using the genomic control (GC) method, and all *p*-values were presented without any inflation (λ = 1). The CMplot^1^ R package was used for drawing Manhattan plots.

We used the simpleM method (Gao et al., 2008) for multiple testing corrections. This method works based on the effective number of independent tests. The SimpleM, first, computes the eigenvalues from the pair-wise SNP correlation matrix created with composite LD from the SNP dataset and then infers the effective number of independent tests (Meff_G) through principal component analysis. Once Meff_G is estimated, a standard Bonferroni correction is applied to control for the multiple testing. The number of independent tests calculated in our study was 8,164. Based on the average number of independent tests and the *P*-value cutoff 0.05, we determined 6.12^∗^10^–6^ and 1.67^∗^10^–4^ as genome-wide and chromosome-wide (suggestive) thresholds, respectively.

### Gene Annotation

Some well-known databases including BioMart-Ensembl,^2^ UCSC Genome Browser^3^, and National Center for Biotechnology Information^4^ were used along with the Ovis aries reference genome assembly (Oar_v3.1) to identify candidate genes within a window of 300 kb up and downstream of the significant SNP.

### Gene-Set Enrichment Analysis

We performed gene-set enrichment analysis in three steps: (i) the assignment of SNP to the known genes, (ii) the assignment of genes to functional categories, (iii) the association analysis between each functional category and the studied traits.

For each trait, an arbitrary threshold of *P*-value ≤ 0.05 was applied to determine significant SNP (based on the results of the pGWAS) for enrichment analysis. The Bioconductor R package biomaRt (Durinck et al., 2005, 2009) and the Oar_v3.1 ovine reference genome assembly were used for flagging genes by significant SNP. The SNPs were assigned to genes if they were within the genomic region or 15 kb upstream or downstream of an annotated gene. Genes harboring at least one significant SNP were considered as significantly associated genes.

The Gene Ontology (GO) database (Ashburner et al., 2000) was used for defining the functional sets of genes. The GO database classifies genes into three functional categories (biological process, molecular function, and cellular component) based on their common properties. Finally, the significant association of a particular GO term with maternal composite traits was calculated using Fisher’s exact test based on the hypergeometric distribution. The *P*-value of the *g* significant genes in the term was computed using the following formula,

$$P-v\,a\,l\,u\,e=1-\sum_{i=0}^{g-1}\frac{\left(\begin{array}{c} s\\ i \end{array}\right)\,\left(\begin{array}{c} m-s\\ k-i \end{array}\right)}{\left(\begin{array}{c} M\\ k \end{array}\right)}$$

where *s* is the total number of significant genes associated with a given maternal composite trait at birth or weaning, *m* is the total number of analyzed genes, and *k* is the total number of genes in the term under consideration (Han and Peñagaricano, 2016). The GO enrichment analysis was performed using the R package goseq (Young et al., 2010). GO terms with more than 5 and less than 500 genes were tested. Functional categories with a nominal *P*-value less than or equal to 0.01 (*p* ≤ 0.01) were considered as significant categories. The ggplot2 (Wickham, 2016) R package was used to visualize the GO analysis results as dot plots.

## Results

### Estimates of Genetic Parameters

Estimates of variance components, heritability (h^2^), and repeatability (R) for the traits are shown in Table 2. Heritability estimates of the traits ranged from 0.08 in TLWW to 0.25 in LMWB. These estimates were in the range reported by previous authors (Rosati et al., 2002; Vatankhah et al., 2008; Mokhtari et al., 2010; Rashidi et al., 2011; Mohammadi et al., 2013; Yavarifard et al., 2015; Abdoli et al., 2019). Clearly, birth weight traits show greater heritability values than weaning weight traits and indicate that maternal genes have bigger effects on the fetus than on the born lamb.

**TABLE 2:** Variance components and genetic parameter estimates for composite reproductive traits in Baluchi sheep.

Trait	$\sigma_{a}^{2}$	$\sigma_{p\,e}^{2}$	$\sigma_{e}^{2}$	$\sigma_{P}^{2}$	*h*^2^±*SE*	*R*±*SE*	EBVs range*	Accuracies avg (sd)**
PBW	0.09	0.03	0.28	0.41	0.23 ± 0.04	0.31 ± 0.02	−0.52 ± 0.51	0.73 (0.06)
TLWB	0.14	0.04	0.43	0.61	0.22 ± 0.04	0.29 ± 0.02	−0.70 ± 0.73	0.73 (0.05)
LMWB	0.10	0.02	0.27	0.39	0.25 ± 0.04	0.30 ± 0.02	−0.56 ± 0.51	0.76 (0.05)
PWW	2.38	2.66	14.84	19.88	0.12 ± 0.04	0.25 ± 0.02	−1.73 ± 2.12	0.60 (0.06)
TLWW	1.76	1.86	19.60	23.23	0.08 ± 0.03	0.15 ± 0.02	−1.41 ± 1.65	0.50 (0.05)
LMWW	1.86	1.25	11.60	14.72	0.13 ± 0.04	0.21 ± 0.02	−2.01 ± 1.69	0.60 (0.06)

*PBW, Progeny Birth Weight; TLWB, Total Litter Weight at Birth; LMWB, Litter Mean Weight at Birth; PWW, Progeny Weaning Weight; TLWW, Total Litter Weight at Weaning; LMWW, Litter Mean Weight at Weaning;*$\sigma_{a}^{2}$, *additive genetic variance;*$\sigma_{p\,e}^{2}$, *permanent environmental variance;*$\sigma_{e}^{2}$, *residual variance*; $\sigma_{p}^{2}$, phenotypic variance; h^2^, heritability; R, repeatability; SE, standard error; *Estimated breeding values range in genotyped animals. **Average and standard deviation of accuracies for genotyped animals.

### GWAS Analysis of the Composite Traits at Birth

**FIGURE 1:**
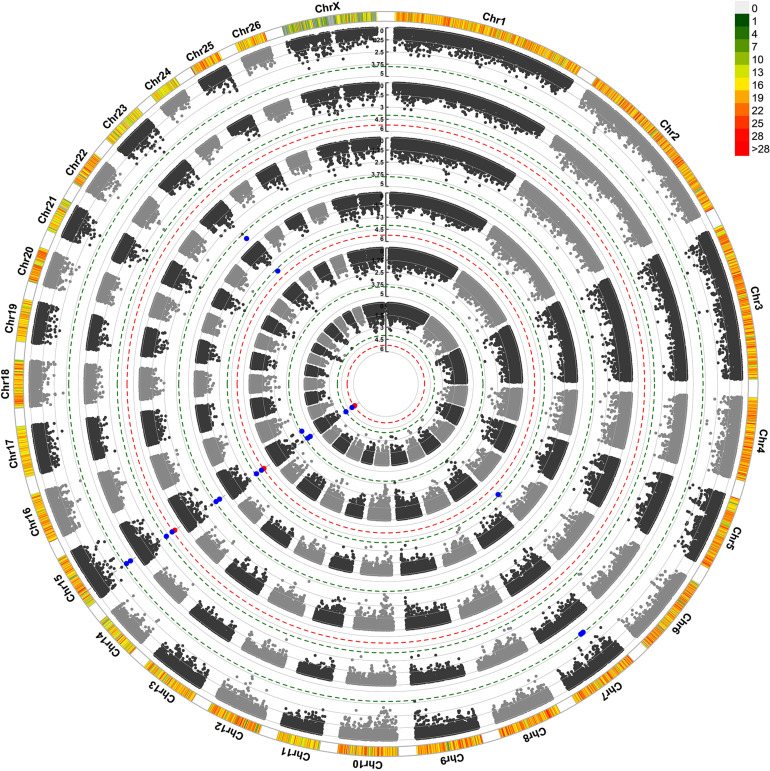
Circular Manhattan plot for associations of SNP with ewe composite traits at birth by two GWAS approaches. The 6 circles from outside to inside represent Progeny Birth Weight (PBW): pGWAS and eGWAS; Total Litter Weight at Birth (TLWB): pGWAS and eGWAS; Litter Mean Weight at Birth (LMWB): pGWAS and eGWAS. The outermost circle shows SNP density in the 1 Mb window for each chromosome. *X*-axis: SNP positions on chromosomes, *Y*-axis: –Log10 *P*-value. The red and blue dashed lines indicate the thresholds for genome-wide (1.22*10^–5^) and chromosome-wide (*P* < 1.67*10^–4^) statistical significance, respectively. The red and blue dots show associated and suggestive SNPs, respectively. pGWAS: GWAS using phenotypes as a response variable; eGWAS: GWAS using EBVs as a response variable.

For maternal composite traits at birth, we searched for maternal genes and pathways that influence the progeny’s birth weight during pregnancy. The results of GWAS analysis are shown in a Circular Manhattan plot in Figure 1. After FDR correction using the simpleM method, one significant and eight suggestive SNPs were identified for ewe’s reproductive traits at birth (Table 3). These SNPs are located on chromosomes OAR6, OAR7, OAR15, and OAR23.

**TABLE 3:** Suggested and associated SNPs with ewe composite traits at birth in Baluchi sheep.

Chr	SNP	Position	Genes in 300 kb interval	Analysis method	Adjusted *P*-value	Trait(s)	MAF
15	**rs422482383**	20,125,491	RDX, FDX1, **ARHGAP20**	pGWAS and eGWAS	2.06 × 10^–5^ 3.34 × 10^–6^	PBW, TLWB and LMWB	0.11
15	rs423274340	20,304,472	ARHGAP20	pGWAS and eGWAS	6.43 × 10^–5^ 8.20 × 10^–6^	PBW, TLWB and LMWB	0.10
15	rs428350449	19,740,174	ZC3H12C, RDX, FDX1	eGWAS	6.34 × 10^–5^	PBW, TLWB and LMWB	0.12
15	rs427207318	69,550,331	Without gene	pGWAS	8.43 × 10^–5^	LMWB	0.08
7	rs408063438	30,207,038	Without gene	pGWAS	9.37 × 10^–5^	PBW	0.01
7	rs399067974	32,062,261	ZCRB1, THBS1, FSIP1	pGWAS	9.37 × 10^–5^	PBW	0.01
7	rs400684038	35,113,511	ZNF106, SNAP23, LRRC57, HAUS2, STARD9, CDAN1, **TTBK2**, UBR1, TMEM62	pGWAS	9.37 × 10^–5^	PBW	0.01
23	rs430043751	46,167,308	EPG5, PSTPIP2, ATP5F1A, HAUS1, RNF165, LOXHD1	pGWAS and eGWAS	2.52 × 10^–5^ 5.21 × 10^–5^	TLWB	0.06
6	rs426428997	109,147,722	Without gene	eGWAS	7.20 × 10^–5^	TLWB	0.05

**Chr, Chromosome number; pGWAS, GWAS using phenotypes as response variable; eGWAS, GWAS using EBVs as response variable; PBW, Progeny Birth Weight; TLWB, Total Litter Weight at Birth; LMWB, Litter Mean Weight at Birth; MAF, Minor allele frequency.* P-values are presented only for the first trait in the Trait(s) column. P-values are adjusted based on the Genomic Control value. Genes with boldface indicate that the significant SNP is located within the gene. SNPs with boldface are significantly associated with the genes but otherwise remain as suggestive SNPs. The regression coefficient of each SNP is provided in Supplementary Table 14.*

Three SNPs including rs422482383, rs423274340, and rs428350449 were identified on OAR15 (19.7–20.3 Mb) which harbors four genes, *RDX*, *FDX1*, *ZC3H12C*, and *ARHGAP20*. The SNPs rs422482383 and rs423274340 were significantly associated with all three traits at birth in both GWAS approaches. The rs422482383 is located within the intron 5 of the *ARHGAP20* gene. Another identified SNP (rs427207318) on OAR15 had a suggestive association with the LMWB trait but did not contain any genes in the 300 kb flanking regions. In addition, we found three marginally suggestive SNPs on OAR7 (rs408063438, rs399067974, and rs400684038) at a distance of 30.2–35.1 Mb. Our BLAST search identified 12 genes in this region, while the SNP rs400684038 was located within the intron 8 of the *TTBK2* gene. Moreover, two SNPs (rs430043751 and rs426428997) were located on OAR23 and OAR6 as they had a suggestive association with TLWB trait.

The SNP rs430043751 on OAR23 was identified in both GWAS approaches and was related to nearly six genes in a 300 kb span, including *EPG5*, *PSTPIP2*, *ATP5F1A*, *HAUS1*, *RNF165*, and *LOXHD1*. This SNP was very close to the threshold line for PBW and LMWB traits in both GWAS approaches. The SNP rs426428997 on the OAR6 did not contain any genes in the searched region.

### GWAS Analysis of the Composite Traits at Weaning

**FIGURE 2:**
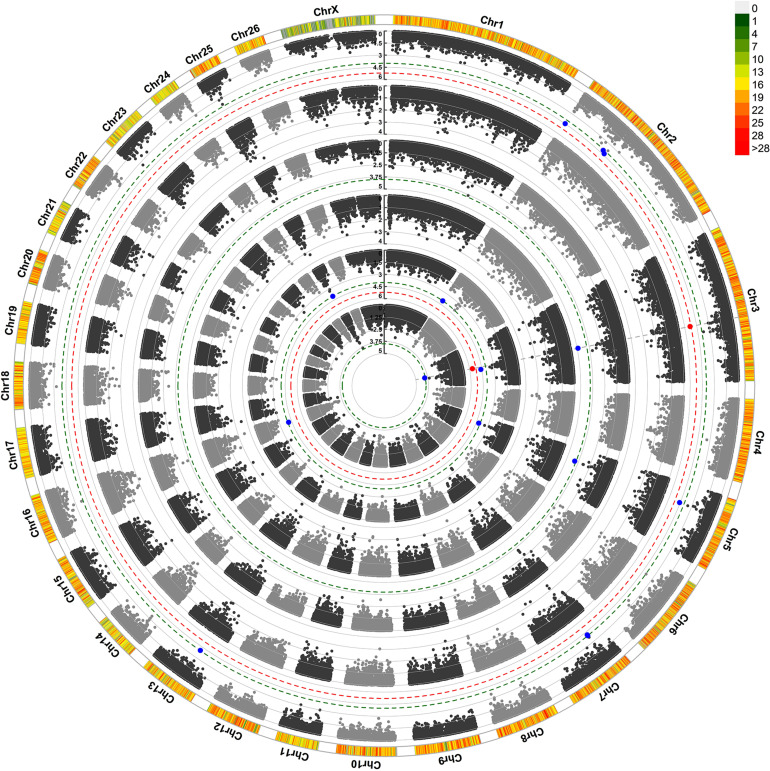
Circular Manhattan plot for associations of SNP with ewe composite traits at weaning by two GWAS approaches. The 4 circles, from outside to inside, represent Progeny Weaning Weight (PWW): pGWAS and eGWAS; Total Litter Weight at Weaning (TLWW): pGWAS and eGWAS; Litter Mean Weight at Weaning (LMWW): pGWAS and eGWAS. The outermost circle shows SNP density in a 1 Mb window for each chromosome. *X*-axis: SNP positions on chromosomes, *Y*-axis: –Log10 *P*-value. The red and blue dashed lines indicate the thresholds for genome-wide (1.22*10^–5^) and chromosome-wide (*P* < 1.67*10^–4^) statistical significance, respectively. The red and blue dots show associated and suggestive SNPs, respectively. pGWAS: GWAS using phenotypes as response variable; eGWAS: GWAS using EBVs as a response variable.

For maternal composite traits at weaning, we looked for maternal genes and pathways that influence the progeny’s weaning weight. The circular Manhattan plot shows associations of SNP markers with traits for both GWAS approaches (Figure 2). After FDR correction (0.05) using the simpleM method, a total of 11 SNPs were significantly and suggestively associated with the maternal composite traits at weaning (Table 4). These SNPs were located on OAR2, OAR3, OAR5, OAR7, OAR13, OAR16, and OAR25. The results of the two GWAS approaches showed similar profiles with one common significant SNPs on OAR3 (rs428404187).

**TABLE 4:** Suggested and associated SNPs with ewe composite traits at weaning in Baluchi sheep.

Chr	SNP	Position	Genes in 300 kb interval	Analysis method	Adjusted *P*-value	Trait(s)	MAF
3	**rs428404187**	131255497	NDUFA12, NR2C1, FGD6, **VEZT**, MIR331, METAP2	pGWAS and eGWAS	4.72 × 10^–6^ 8.60 × 10^–5^	PWW, TLWW,and LMWW	0.03
5	rs398620273	32383306	HSD17B4, DMXL1, **DTWD2**	pGWAS	2.72 × 10^–5^	PWW, TLWW, and LMWW	0.28
2	rs412011189	1426911	EIPR1	pGWAS	3.39 × 10^–5^	PWW and LMWW	0.2
2	rs411656768	81968762	NFIB	pGWAS	4.74 × 10^–5^	PWW	0.11
7	rs430218107 rs419540936	23778602 23939664	EDDM3B, ANG1, RNASE1, RNASE6, RNASE4, ANG2, UQCRFS1, RNASE12, RNASE11, RNASE9, RNASE10, PIP4P1, APEX1, OSGEP, KLHL33, TEP1, PARP2, RPPH1, SNORA79B, CCNB1IP1, TTC5, OR11H4, OR11H7, OR11H6	pGWAS pGWAS	7.42 × 10^–5^ 9.79 × 10^–5^	PWW PWW	0.42 0.29
2	rs403459195	77075145	RPLP0	pGWAS	7.88 × 10^–5^	PWW	0.26
13	rs401393221	30320719	PTER, C1ql3, **RSU1**, CUBN, VIM	pGWAS	9.50 × 10^–5^	PWW	0.29
3	rs404069303	143726957	SNORA62	pGWAS	1.90 × 10^–5^	LMWW	0.04
16	rs409558668	39225407	PRLR, AGXT2, DNAJC21, BRIX1, RAD1, **TTC23L**, RAI14	pGWAS	7.95 × 10^–5^	LMWW	0.23
25	rs405045517	16553906	CDK1, FTH1, **RHOBTB1**	pGWAS	9.89 × 10^–5^	LMWW	0.09

*Chr, Chromosome number; pGWAS, GWAS using phenotype; eGWAS, GWAS using EBVs; PWW, Progeny Weaning Weight; TLWW, Total Litter Weight at Weaning; LMWW, Litter Mean Weight at Weaning; MAF, Minor allele frequency. P-values are presented only for the first trait in the Trait(s) column. P-values are adjusted based on the Genomic Control value. Genes with boldface indicate that significant SNPs are located within the gene. SNPs with boldface are significantly associated with genes but otherwise are suggestive SNPs. The regression coefficient of each SNP is provided in Supplementary Table 15.*

The most significant SNP (rs428404187, *p* = 4.72 × 10^–6^) was located on OAR3 (131.2 Mb) and was significant for the three composite traits at weaning in the pGWAS approach. Besides, this SNP had a suggestive association with LMWW in the eGWAS approach and was found to be located within the intron 1 of the *VEZT* gene. For PWW and TLWW traits, there were no significant or suggestive SNPs using eGWAS. Another common suggestive SNP, rs398620273, and was associated with three composite traits at weaning. This SNP located within the *DTWD2* gene (intron 2) on OAR5. SNP rs412011189 on OAR2 had a suggestive association with PWW and LMWW and was close to the *EIPR1* gene. In addition, we found five suggestively associated SNPs with PWW, including, rs411656768 and rs403459195 on OAR2, rs430218107 and rs419540936 on OAR7, and rs401393221 on OAR13. The SNPs on OAR7 harbor 24 genes in a 300 Kb span, seven of which were RNase genes. SNP on OAR13 was located in the *RSU1* gene (intron 2), while the *VIM* gene is located downstream of this SNP at a distance of 4.2 kb. Three suggestive SNPs were found to be related to LMWW and were identified on OAR3 (rs404069303), OAR16 (rs409558668), and OAR25 (rs405045517). The SNP on OAR16 was near seven genes in a 300 kb span (*PRLR*, *AGXT2*, *DNAJC21*, *BRIX1*, *RAD1*, *TTC23L*, and *RAI14*). This SNP was located in the ovine gene *TTC23L* (intron 2). Additionally, the *Prolactin receptor* (*PRLR*) gene was located close to this SNP. Another suggestive SNP (rs405045517) was located on OAR25 and harbored three genes (*CDK1*, *FTH1*, and *RHOBTB1*) in the searched region. This SNP was located within the *RHOBTB1* gene (intron 4). Also, the *FTH1* gene was found to be located close to this SNP.

### Gene-Set Enrichment Analysis

The results of GWAS were complemented with gene set enrichment analysis using the GO database. In total, 23,462 of the 45,342 SNPs being tested in the GWAS, were located within or 15 kb upstream or downstream of 15,815 annotated genes in the Oar.v3.1 ovine genome assembly. On average, 1,310 out of the 15,815 genes (ranging from 1,291 for LMWW to 1,351 for TLWW) contained at least one significant SNP (*P*-value ≤ 0.05) and were defined as significantly associated with maternal composite traits. GO terms with a nominal *P*-value ≤ 0.01 were reported as significant terms. GWAS results using direct phenotypes (pGWAS) were used for analysis and each trait was analyzed separately.

### Gene-Set Enrichment Analysis of the Composite Traits at Birth

**FIGURE 3:**
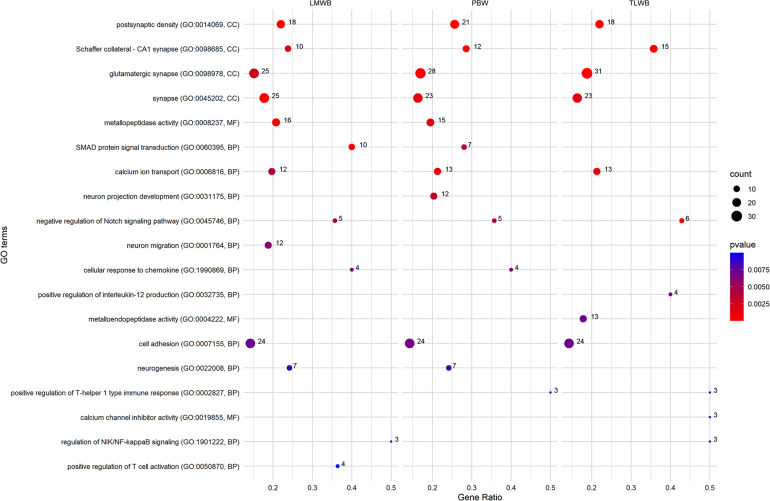
Most related Gene Ontology (GO) terms were significantly (*p* ≤ 0.01) enriched using genes associated with maternal composite traits at birth. PBW, Progeny Birth Weight; TLWB, Total Litter Weight at Birth; LMWB, Litter Mean Weight at Birth. Gene Ratio: Number of the significant genes in the category/Number of all genes in the category. Complete associated GO terms with these traits are provided in Supplementary Tables 1–3.

Figure 3 shows a set of GO terms that were significantly (*P* ≤ 0.01) enriched with genes associated with maternal composite traits at birth. Several GO terms related to the neural system, showed an overrepresentation of significant genes, including *postsynaptic density* (GO:0014069), *Schaffer collateral-CA1 synapse* (GO:0098685), *glutamatergic synapse* (GO:0098978), *synapse* (GO:0045202), *neuron projection development* (GO:0031175), *neurogenesis* (GO:0022008), and many other terms that were not included in Figure 3 (see Supplementary Tables 1–3). The *calcium ion transport* (GO:0000045) was associated with all composite traits at birth. Furthermore, *calcium channel inhibitor activity* (GO:0019855) showed an overrepresentation of significant genes associated with TLWB. Many GO terms related to the immune system also showed significant enrichment of genes associated with composite traits at birth, including *cellular response to chemokine* (GO:1990869), *positive regulation of T-helper 1 type immune response* (GO:0002827), *positive regulation of interleukin-12 production* (GO:0032735), and *positive regulation of T cell activation* (GO:0050870). Several significant GO terms were related to the signaling process. In particular, *SMAD protein signal transduction* (GO:0060395), *negative regulation of Notch signaling pathway* (GO:0045746), and *regulation of NIK/NF-kappaB signaling* (GO:1901222) showed an overrepresentation of significant genes. In addition, we identified *cell adhesion* (GO:0007155) and *metallopeptidase activity* (GO:0008237) GO terms as significant processes that were associated with the composite traits at birth. Several other GO terms were also significant in terms of composite traits at birth. The full list is provided in Supplementary Tables 1–3.

### Gene-Set Enrichment Analysis of the Composite Traits at Weaning

**FIGURE 4:**
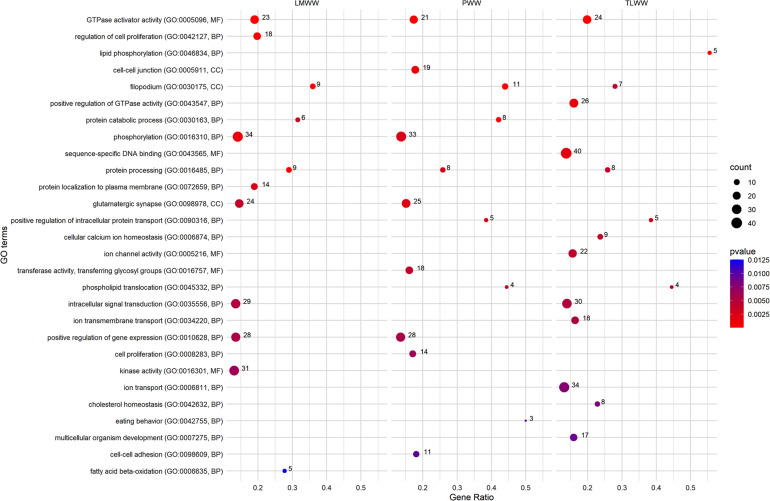
Most related Gene Ontology (GO) terms were significantly enriched (*p* ≤ 0.01) using genes associated with ewe composite traits at weaning. PWW: Progeny Weaning Weight; TLWW, Total Litter Weight at Weaning; LMWW, Litter Mean Weight. Gene Ratio: Number of the significant genes in the category/Number of all genes in the category. Complete associated GO terms with these traits are provided in Supplementary Tables 4–6.

Figure 4 shows a set of GO terms that were significantly (*p* ≤ 0.01) enriched by genes associated with weaning traits. The *Filopodium* (GO:0030175) term was significantly associated with the composite traits at weaning. Moreover, multiple GO terms were linked to protein metabolism, including *protein catabolic process* (GO:0030163), *positive regulation of intracellular protein transport* (GO:0090316), *protein processing* (GO:0016485), and *protein localization to plasma membrane* (GO:0072659). Several GO terms related to GTPase activity were recognized as significant. Among these, *GTPase activator activity* (GO:0005096) showed an overrepresentation of significant genes associated with all composite traits at weaning. Many significant GO terms were related to ion transport and homeostasis and channel activity, including *cellular calcium ion homeostasis* (GO:0006874), *ion channel activity* (GO:0005216), *ion transmembrane transport* (GO:0034220), and *ion transport* (GO:0006811). On the other hand, many GO terms that were related to the metabolism of lipids, cholesterol, and fatty acids showed an overrepresentation of genes associated with the traits at weaning, including *phospholipid translocation* (GO:0045332), *lipid phosphorylation* (GO:0046834), *cholesterol homeostasis* (GO:0042632), and *fatty acid beta-oxidation* (GO:0006635). In addition, several terms were related to cell proliferation (GO:0008283 and GO:0042127), gene expression (GO:0010628), cell adhesion (GO:0098609), cell junction GO:0005911), Protein kinase activity (GO:0016301), and phosphorylation (GO:0016310). The full list of associated terms with weaning weight traits is provided in Supplementary Tables 4–6.

## Discussion

### GWAS and GSEA of Composite Traits at Birth

Here, we performed a whole-genome scan on Iranian Baluchi sheep for six maternal composite traits. We regressed lambs’ weights at birth and weaning on ewes’ genotype and tried to identify gene variants (or regions) in the genome of ewes that affect pregnancy outcome (birth weights) and weaning weights of the lambs. To our knowledge, this is the second GWAS on these traits in sheep. In the first study, Abdoli et al. (2019) reported five genes neighboring the top SNP (on OAR2, OAR3, OAR15, and OAR16), including *TEX12*, *BCO2*, *WDR70*, *INHBE*, and *INHBC* as possible candidate genes affecting composite traits of the Lori–Bakhtiari sheep. In this study, to attain more consistent findings, two different GWAS approaches were used. Both approaches identified similar regions that may explain some part of the genetic variation in the studied traits. We identified four genes on OAR15 (19.7–20.3 Mb), namely, *RDX*, *FDX1*, *ZC3H12C*, and *ARHGAP20* as maternal genes affecting composite traits at birth. *RDX* (Radixin) is part of the ERM (*EZR-RDX-MSN*) cytoskeleton linker protein family. The expression of ERM proteins in the blastocyst and the uterus has been reported and linked to implantation potential in mice (Matsumoto et al., 2004). *Ferredoxin* (*FDX1*) is an electron transport intermediate that is functional in mitochondrial cytochromes P450 and is found mainly in steroidogenic tissues like testis, adrenal glands, ovaries, and placenta (Sheftel et al., 2010). In this study, the *ARHGAP20* gene was identified as the candidate gene in both GWAS approaches. High expression levels of the *ARHGAP20* gene in the brain have been reported, indicating a role by this gene in neurogenesis (Kalla et al., 2005). The *Zc3h12c* is an endogenous inhibitor of TNFα-induced inflammatory signaling in the human umbilical vein and endothelial cells. It seems that the *Zc3h12c* gene plays a role in immune regulation in pregnancy (Liu et al., 2013). Abdoli et al. (2019) identified an SNP on OAR15 located on 22.02 Mb significantly associated with the TLWB trait, which is close to the region identified in this study and reinforces this possibility that this region on OAR15 is likely to affect fetal development during pregnancy.

Our GWAS analysis identified a region on OAR7 at 30.2–35.1 Mb that contains three suggestively associated SNPs with PBW. This region harbors 12 genes, such as *THBS1* and *TTBK2*. It has been reported that the expression of *THBS1* by placental cells is crucial for the formation of the placental structure (Ostankova et al., 2011). One of these SNPs, rs400684038, is located within the *TTBK2* gene. The *TTBK2* gene encodes a serine-threonine kinase that phosphorylates tau and tubulin proteins and is a critical regulator of processes that initiate the assembly of primary cilia in the embryo (Goetz et al., 2012). Both GWAS approaches identified the SNP rs430043751 on OAR23 associated with TLWW. This SNP harbors six genes, including *EPG5*, *PSTPIP2*, *ATP5F1A*, *HAUS1*, *RNF165a*, and *LOXHD1.* The *EPG5* gene encodes a protein with a crucial role in the autophagy pathway which contributes to early differentiation in human embryonic stem cells (Tra et al., 2011).

**FIGURE 5:**
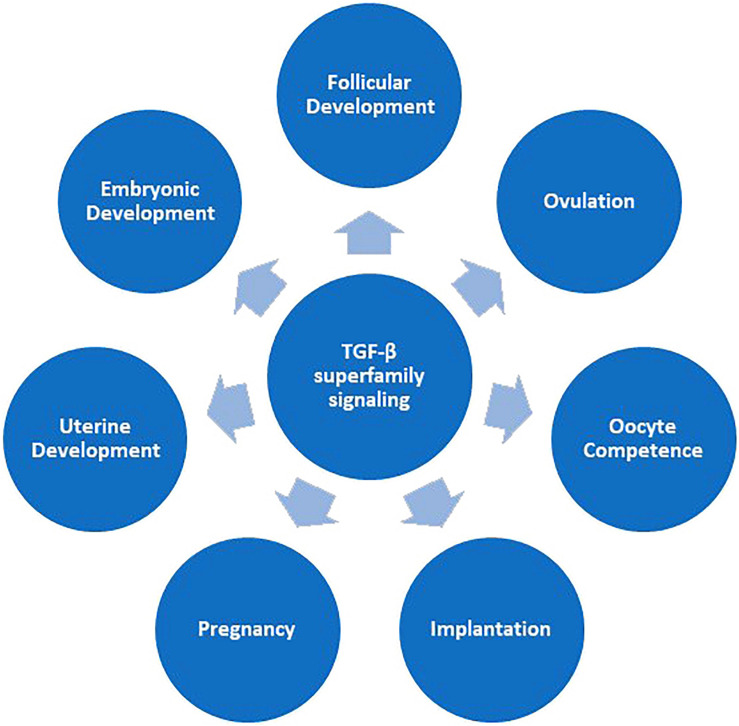
Main functions of TGF-β family signaling in female reproduction (Li, 2014). SMAD proteins transduce signals from TGF-β superfamily ligands.

Our gene set analysis identified several significantly associated GO terms with maternal composite traits at birth (Figure 3). Interestingly, many GO terms were related to the neural system and showed an overrepresentation of significant genes. Numerous studies have reported that neural alterations occur extensively in pregnant women’s brains (Cohen and Mizrahi, 2015; Bridges, 2016). Notably, our identified gene, *ARHGAP20*, has a role in neurogenesis. We identified two pathways (GO:0000045 and GO:0019855) related to calcium ion metabolism. A report suggests that placental calcium transfer increases during pregnancy to match fetal needs and ensure appropriate fetal skeletal mineralization (Strid and Powell, 2000). However, recent evidence has grown inconsistent about the effects of maternal calcium on birth weight (Thompson et al., 2019). In this regard, Imdad and Bhutta (2012) concluded that calcium supplementation during pregnancy is associated with a reduction in risk of gestational hypertensive disorders and pre-term birth and an increase in birth weight. The *positive regulation of T-helper 1 type immune response* term was significant for all three composite traits at birth. During pregnancy, the fetal expression of paternal major histocompatibility (MHC) antigens renders it foreign, and thus, the maternal immune system must tolerate the semi-allogeneic fetus to support the pregnancy, without causing the mother to become susceptible to infection (Munoz-Suano et al., 2011). A shift in the balance of T_Helper_ (T_H1_)/T_H2_ cytokine production by maternal peripheral blood leukocytes is regarded as a commonly important feature of successful mammalian pregnancy (Wattegedera et al., 2008). Recently, it has been reported that pregnancy can change the production of Th1 and Th2 cytokines in the maternal thymus in sheep (Zhang et al., 2019). GO terms related to the signaling pathways also showed an overrepresentation of significant genes. *SMAD protein signal transduction* (GO:0060395) was one of these pathways. SMAD proteins transduce signals from the TGF-β superfamily ligands and, as a result, regulate target gene expression. TGF-β superfamily signaling is vital for female reproduction (Figure 5).

It has been reported that SMAD proteins have roles in maintaining the structural and functional integrity of the oviduct and uterus. They are essential for the establishment and maintenance of pregnancy (Rodriguez et al., 2016). Another signaling pathway is Notch signaling which exerts effects throughout the pregnancy and plays an important role in placental angiogenesis, normal placental function, and trophoblast function (Zhao and Lin, 2012). The *NIK/NF-kappaB signaling* (GO:1901222) works as a transcription factor involved in inflammatory and immune responses (Baldwin, 1996). The effects of NF-κB and its signaling pathway on the human myometrium during pregnancy and parturition are well-reviewed (Cookson and Chapman, 2010).

### GWAS and GSEA of Composite Traits at Weaning

Genes, including *NDUFA12*, *NR2C1*, *FGD6*, *VEZT*, *MIR331*, and *METAP2* were identified on OAR3, specifically around the SNP rs428404187. This SNP is significantly associated with all composite traits at weaning and located within the *VEZT* gene which has a major role in the cellular adhesion process. The cell adhesion process has a widespread effect on the development of mammary glands. It mainly occurs in late pregnancy and partially in the onset of lactation (Shamir and Ewald, 2015). Notably, we identified the *cell-cell adhesion* (GO:0098609) pathway as a significant GO term associated with the PWW trait in our gene set analysis. Another gene in this region, *NR2C1*, is a nuclear steroid hormone receptor. This gene acts as a transcription factor and plays an important role in the differentiation of mammary glands and its development in late pregnancy and during lactation (To and Andrechek, 2018).

The SNP rs398620273 on OAR5 was suggestively associated with all three composite traits at weaning. It is located within the *DTWD2* gene and is likely to be involved in RNA processing (Burroughs and Aravind, 2014). The *HSD17B4* is another gene that was identified around this SNP and plays an important role in feed intake and food conservation (Salleh et al., 2017). In dairy cows, feed intake is a major factor that controls milk production in high-yielding dairy cows in early lactation (Johansen et al., 2018). We identified the *RPLP0* gene on OAR2. Dominant expressions of RPLP0 occur in mammary vasculature tissues during lactation (Lee et al., 2010). The *SNORA62* gene was identified on OAR3 as a suggestive gene affecting the LMWW trait. Recently, a GWAS of milk fatty acid composition led to the identification of the *SNORA62* gene as a candidate gene affecting fatty acid content in milk (Palombo et al., 2018).

We identified 24 suggestive genes on OAR7 related to PWW and seven of which belong to the pancreatic ribonuclease A family (*RNases*). It seems that this region plays an important role in RNA processing. The *RNASE5* is known as a functional gene in milk production, specifically in milk protein percentage (Raven et al., 2013). We identified a suggestive SNP, rs401393221, within the RSU1 gene on OAR13. Using meta-analysis and supervised machine learning models on microarray data, the *RSU1* gene has been identified as DEG during the lactation process in both approaches (Farhadian et al., 2018). It is worth noting that the *RSU1* gene is a member of the *milk proteins* KEGG pathway. In addition, we identified *CUBN* and *VIM* genes around this SNP on OAR13. Association between *CUBN* gene and variation in vitamin B-12 content in bovine milk have been reported (Rutten et al., 2013). *VIM* is a cytoskeletal type III intermediate and has a critical role in the development of mammary glands (Peuhu et al., 2017). A fourfold increase of *VIM* protein in lactating tissues compared to resting tissues reported (Michalczyk et al., 2001).

The SNP rs409558668 was identified on OAR16 with a suggestive association with the LMWW trait. This SNP is located within the *TTC23L* gene and harbors the *PRLR* (Prolactin receptor) gene. The *TTC23L* gene is highly expressed during lactation (Paten, 2014) and is identified as a candidate gene that can affect mastitis in Holstein cows (Tiezzi et al., 2015). The *PRLR* gene is usually expressed in lactating mammary glands and it has been shown that the polymorphism in exon 3 and 7 of the *PRLR* gene is correlated with milk production in Holstein cows (Zhang et al., 2008). Also, we identified the *FTH1* gene on OAR16. The *FTH1* gene encodes the heavy subunit of ferritin. The presence of ferritin in cow’s and buffalo’s milk has been reported (Farhadian et al., 2018; Arora et al., 2019).

Through gene set analysis for composite traits at weaning, several maternal functional categories were identified. Many GO terms were discovered in association with protein metabolism, protein transport, and fatty acid metabolism. Recently, in a transcriptome analysis study on buffalo milk, the *protein metabolism* (GO:0019538) pathway was identified as a significant term (Arora et al., 2019). Considerable changes occur in the amount of fatty acid synthesis during late pregnancy and lactation. These changes have been reported in a variety of species, like rats, rabbits, pigs, and cows (Larson and Smith, 1974; Abdollahi-Arpanahi et al., 2019). There have been significant observations regarding GO terms linked to calcium and ions metabolism and transport. Many different comparative transcriptome analyses have reported the role of calcium metabolism-related pathways in the lactation process (Arora et al., 2019; Bhat et al., 2019). In the current study, *Phosphorylation* (GO:0016310) was identified as a significant pathway associated with PWW and LMWW traits. On average, caseins comprise 80% of proteins in cow and sheep milk, so, phosphorylation by the Casein Kinase enzyme is a crucial step for milk production in the lactating mammary gland (Bionaz et al., 2012). Notably, the *kinase activity* (GO:0016301) pathway is another significant term for LMWW that catalyzes the transfer of a phosphate group to a substrate molecule. The c*ell-cell adhesion* pathway was significantly associated with PWW. The effects of cell adhesion molecules on the lactogenesis process have been reviewed thoroughly in the scientific literature (Morrison and Cutler, 2010). The *VEZT* gene, one of our identified genes in the GWA analysis, is a member of this pathway. The *GTPase activator activity* GO term was identified as a significant pathway related to all three composite traits at weaning. GTPases are known to be involved in numerous secretory processes and play an important role in the translation and translocation of proteins, the secretion of milk fat globules, and, probably, other milk components (Arora et al., 2019). Many GO terms associated with cell proliferation and differentiation were also detected as significant. Most mammary growth takes place through pregnancy. Mammary gland cell proliferation and differentiation have a great impact on milk yield and lactation persistency (Davis, 2017). Mammary epithelial cells (MECs) are key cells that are present in lactating mammary glands and are responsible for milk production. The number of MECs in the mammary gland and their secretory activity are crucial factors that regulate milk yield (Herve et al., 2016). Milk is essential for lamb survival and growth in the first 3–4 weeks of life and 70% of the difference in weight gain from 3 to 12 weeks is attributed to milk intake (Morgan et al., 2007). In twin lambs, prenatal ewe traits (e.g., ewe weight at mating and set stocking, as well as ewe body condition score at mating and stocking) usually have minimal effects on milk yield and lamb growth until the time of weaning, but milk yield and composition have the greatest proportion of variation in lamb weight gain (Danso et al., 2016) which is consistent with the results of this study.

To date, this is the second GWAS report on composite reproductive traits in sheep. At this point, these genes are merely candidates. The identification of validated causal genetic variants that underlie production traits is one of the main challenges in current livestock genetic research. It is important to point out that the limited number of animals and low to moderate heritability of the traits, actually hinder the detection of strong association signals. Composite traits are complex and try to capture growth, yield, and several different aspects of fertility into a combined selection of traits at the same time. As such, the traits are necessarily polygenic by far in nature and it will require thousands of genotypes to disentangle true signals from background noise. In this study, a good amount of caution was considered for performing analysis and reporting the results. We used two GWAS approaches to confirm the results and reported the *p*-values without any inflation to avoid false positives as much as possible. Of course, we couldn’t apply a stringent threshold for all reported SNPs and, hopefully, there are no false positives in the results. However, the Baluchi sheep is a widely used breed in east Iran, but it is not widely distributed in other parts of the world. Finally, while this study does improve our understanding of an interesting but less characterized breed, it will still be useful to see if these results can be broadly applicable to other breeds as well.

## Conclusion

We used GWAS and GSEA together to find genes and pathways affecting maternal composite traits at birth and weaning in sheep. Several genes including *RDX*, *FDX1*, *ARHGAP20*, *ZC3H12C*, *THBS1*, and *EPG5* were associated with composite traits at birth. These genes play roles in pregnancy, particularly in autophagy, immune response, angiogenesis, and placental formation. Gene set analysis identified *calcium ion transport* as a significant GO term that affecting composite traits at birth. In addition, we identified many genes (e.g., *NR2C1*, *VEZT*, *HSD17B4*, *RSU1*, *CUBN*, *VIM*, *PRLR*, and *FTH1*) as genes affecting composite traits at weaning. Our gene set analysis on these traits identified several significantly related GO terms, e.g., protein processing and transport, phospholipid translocation, ion transport, and cell-cell adhesion. As expected, most identified genes and GO terms have a role in milk production or in mammary gland development, which means that feeding lambs by milk can have the greatest impact on weight gain as compared to other effects of maternal origin. This suggests that farmers should select ewes with higher milk yields to maximize lamb growth until weaning. Moreover, the results provide a good insight into how maternal genes and pathways influence progeny weight at birth and, subsequently, at weaning.

## Data Availability Statement

The datasets presented in this study can be found in online repositories. The names of the repository/repositories and accession number(s) can be found below: https://figshare.com/ and https://doi.org/10.6084/m9.figshare.11859996.v1.

## Ethics Statement

The animal study was reviewed and approved by the Sari Agricultural Sciences and Natural Resources University.

## Author Contributions

SME-F: conceptualization, data curation, methodology, formal analysis, software, visualization, writing—original draft, and writing—review and editing. MG: methodology, supervision, resources, and writing—review and editing. SH: project administration, supervision, and writing—review and editing. RA-A: methodology, supervision, and writing—review and editing. All authors contributed to the article and approved the submitted version.

## Conflict of Interest

The authors declare that the research was conducted in the absence of any commercial or financial relationships that could be construed as a potential conflict of interest.

## Publisher’s Note

All claims expressed in this article are solely those of the authors and do not necessarily represent those of their affiliated organizations, or those of the publisher, the editors and the reviewers. Any product that may be evaluated in this article, or claim that may be made by its manufacturer, is not guaranteed or endorsed by the publisher.

## Footnotes

1
https://github.com/YinLiLin/R-CMplot

2
www.ensembl.org/biomart

3
http://genome.ucsc.edu

4
https://www.ncbi.nlm.nih.gov

## Supplementary Material

Supplementary Tables 1–6 List of significantly (*p* ≤ 0.01) associated GO terms with the studied traits.

Supplementary Table 7 Correlations between the original traits and EBVs of the traits.

Supplementary Tables 8–13 List of significant genes used for GSEAs.

Supplementary Table 14 The regression coefficient (SNP effects) of reported SNPs for composite traits at birth.

Supplementary Table 15 The regression coefficient (SNP effects) of reported SNPs for composite traits at weaning.

## References

[B1] Abbasi M. A. Abdollahi-Arpanahi R. Maghsoudi A. Torshizi R. V. Nejati-Javaremi A. (2012). Evaluation of models for estimation of genetic parameters and maternal effects for early growth traits of Iranian Baluchi sheep. *Small Rumin. Res.* 104 62–69. 10.1016/J.SMALLRUMRES.2011.10.003

[B2] Abdoli R. Mirhoseini S. Ghavi Hossein-Zadeh N. Zamani P. Ferdosi M. Gondro C. (2019). Genome-wide association study of four composite reproductive traits in Iranian fat-tailed sheep. *Reprod. Fertil. Dev.* 31 1127–1133. 10.1071/RD18282 30958977

[B3] Abdoli R. Mirhoseini S. Z. Ghavi Hossein-Zadeh N. Zamani P. Gondro C. (2018). Genome-wide association study to identify genomic regions affecting prolificacy in Lori-Bakhtiari sheep. *Anim. Genet.* 49 488–491. 10.1111/age.12700 30079564

[B4] Abdollahi-Arpanahi R. Carvalho M. R. Ribeiro E. S. Peñagaricano F. (2019). Association of lipid-related genes implicated in conceptus elongation with female fertility traits in dairy cattle. *J. Dairy Sci.* 102 10020–10029. 10.3168/jds.2019-17068 31477299

[B5] Arora R. Sharma A. Sharma U. Girdhar Y. Kaur M. Kapoor P. (2019). Buffalo milk transcriptome: a comparative analysis of early, mid and late lactation. *Sci. Rep.* 9:5993. 10.1038/s41598-019-42513-2 PMC6461664 30979954

[B6] Ashburner M. Ball C. A. Blake J. A. Botstein D. Butler H. Cherry J. M. (2000). Gene ontology: tool for the unification of biology. *Nat. Genet.* 25:25. 10.1038/75556 PMC3037419 10802651

[B7] Aulchenko Y. S. Ripke S. Isaacs A. Van Duijn C. M. (2007). GenABEL: an R library for genome-wide association analysis. *Bioinformatics* 23 1294–1296. 10.1093/bioinformatics/btm108 17384015

[B8] Baldwin A. S. Jr. (1996). The NF-κB and IκB proteins: new discoveries and insights. *Annu. Rev. Immunol.* 14 649–681. 10.1146/annurev.immunol.14.1.649 8717528

[B9] Bhat S. A. Ahmad S. M. Ibeagha-Awemu E. M. Bhat B. A. Dar M. A. Mumtaz P. T. (2019). Comparative transcriptome analysis of mammary epithelial cells at different stages of lactation reveals wide differences in gene expression and pathways regulating milk synthesis between Jersey and Kashmiri cattle. *PLoS One* 14:e0211773. 10.1371/journal.pone.0211773 30721247 PMC6363229

[B10] Bionaz M. Hurley W. Loor J. (2012). “Milk protein synthesis in the lactating mammary gland: insights from transcriptomics analyses,” in *Milk protein*, ed. Hurley W. (Rijeka: InTech), 285–324.

[B11] Bridges R. S. (2016). Long-term alterations in neural and endocrine processes induced by motherhood in mammals. *Horm. Behav.* 77 193–203. 10.1016/j.yhbeh.2015.09.001 26388065 PMC4724454

[B12] Bromley C. M. Van Vleck L. D. Snowder G. D. (2001). Genetic correlations for litter weight weaned with growth, prolificacy, and wool traits in Columbia, Polypay, Rambouillet, and Targhee sheep. *J. Anim. Sci.* 79 339–346. 10.2527/2001.792339x 11219442

[B13] Browning B. L. Zhou Y. Browning S. R. (2018). A one-penny imputed genome from next-generation reference panels. *Am. J. Hum. Genet.* 103 338–348. 10.1016/j.ajhg.2018.07.015 30100085 PMC6128308

[B14] Burroughs A. M. Aravind L. (2014). Analysis of two domains with novel RNA-processing activities throws light on the complex evolution of ribosomal RNA biogenesis. *Front. Genet.* 5:424. 10.3389/fgene.2014.00424 25566315 PMC4275035

[B15] Cohen L. Mizrahi A. (2015). Plasticity during motherhood: changes in excitatory and inhibitory layer 2/3 neurons in auditory cortex. *J. Neurosci.* 35 1806–1815. 10.1523/jneurosci.1786-14.2015 25632153 PMC6795264

[B16] Cookson V. J. Chapman R. (2010). NF-kB function in the human myometrium during pregnancy and parturition. *Histol. Histopathol.* 25 945–956. 20503182 10.14670/HH-25.945

[B17] Dadousis C. Pegolo S. Rosa G. J. M. Gianola D. Bittante G. Cecchinato A. (2017). Pathway-based genome-wide association analysis of milk coagulation properties, curd firmness, cheese yield, and curd nutrient recovery in dairy cattle. *J. Dairy Sci.* 100 1223–1231. 10.3168/jds.2016-11587 27988128

[B18] Danso A. S. Morel P. C. H. Kenyon P. R. Blair H. T. (2016). Relationships between prenatal ewe traits, milk production, and preweaning performance of twin lambs. *J. Anim. Sci.* 94 3527–3539. 10.2527/jas.2016-0337 27695783

[B19] Davis S. R. (2017). Triennial lactation symposium/bolfa: mammary growth during pregnancy and lactation and its relationship with milk yield. *J. Anim. Sci.* 95 5675–5688. 10.2527/jas2017.1733 29293774 PMC6292317

[B20] Duguma G. Schoeman S. J. Cloete S. W. P. Jordaan G. F. (2002). Genetic and environmental parameters for ewe productivity in Merinos. *S. Afr. J. Anim. Sci.* 32 154–159.

[B21] Durinck S. Moreau Y. Kasprzyk A. Davis S. De Moor B. Brazma A. (2005). BioMart and bioconductor: a powerful link between biological databases and microarray data analysis. *Bioinformatics* 21 3439–3440. 10.1093/bioinformatics/bti525 16082012

[B22] Durinck S. Spellman P. T. Birney E. Huber W. (2009). Mapping identifiers for the integration of genomic datasets with the R/Bioconductor package biomaRt. *Nat. Protoc.* 4:1184. 10.1038/nprot.2009.97 19617889 PMC3159387

[B23] Esmaeili-Fard S. M. Gholizadeh M. Hafezian S. H. Abdollahi-Arpanahi R. (2021). Genome-wide association study and pathway analysis identify NTRK2 as a novel candidate gene for litter size in sheep. *PLoS One* 16:e0244408. 10.1371/journal.pone.0244408 33481819 PMC7822323

[B24] Farhadian M. Rafat S. A. Hasanpur K. Ebrahimi M. Ebrahimie E. (2018). Cross-species meta-analysis of transcriptomic data in combination with supervised machine learning models identifies the common gene signature of lactation process. *Front. Genet.* 9:235. 10.3389/fgene.2018.00235 30050559 PMC6052129

[B25] Gao X. Starmer J. Martin E. R. (2008). A multiple testing correction method for genetic association studies using correlated single nucleotide polymorphisms. *Genet. Epidemiol.* 32 361–369. 10.1002/gepi.20310 18271029

[B26] Gholizadeh M. Ghafouri-Kesbi F. (2015). Estimation of genetic parameters for growth-related traits and evaluating the results of a 27-year selection program in Baluchi sheep. *Small Rumin. Res.* 130 8–14. 10.1016/j.smallrumres.2015.07.032

[B27] Gholizadeh M. Rahimi-Mianji G. Nejati-Javaremi A. De Koning D. J. Jonas E. (2014). Genomewide association study to detect QTL for twinning rate in Baluchi sheep. *J. Genet.* 93 489–493. 10.1007/s12041-014-0372-1 25189245

[B28] Goetz S. C. Liem K. F. Jr. Anderson K. V. (2012). The spinocerebellar ataxia-associated gene Tau tubulin kinase 2 controls the initiation of ciliogenesis. *Cell* 151 847–858. 10.1016/j.cell.2012.10.010 23141541 PMC3496184

[B29] Han Y. Peñagaricano F. (2016). Unravelling the genomic architecture of bull fertility in Holstein cattle. *BMC Genet.* 17:143. 10.1186/s12863-016-0454-6 27842509 PMC5109745

[B30] Hanford K. J. Van Vleck L. D. Snowder G. D. (2003). Estimates of genetic parameters and genetic change for reproduction, weight, and wool characteristics of Targhee sheep. *J. Anim. Sci.* 81 630–640. 10.2527/2003.813630x 12661643

[B31] Hayes B. J. Bowman P. J. Chamberlain A. C. Verbyla K. Goddard M. E. (2009). Accuracy of genomic breeding values in multi-breed dairy cattle populations. *Genet. Sel. Evol.* 41:51. 10.1186/1297-9686-41-51 PMC2791750 19930712

[B32] Henderson C. R. (1975). Best linear unbiased estimation and prediction under a selection model. *Biometrics* 31 423–447. 10.2307/2529430 1174616

[B33] Herve L. Quesnel H. Lollivier V. Boutinaud M. (2016). Regulation of cell number in the mammary gland by controlling the exfoliation process in milk in ruminants. *J. Dairy Sci.* 99 854–863. 10.3168/jds.2015-9964 26433413

[B34] Imdad A. Bhutta Z. A. (2012). Effects of calcium supplementation during pregnancy on maternal, fetal and birth outcomes. *Paediatr. Perinat. Epidemiol.* 26 138–152. 10.1111/j.1365-3016.2012.01274.x 22742607

[B35] Johansen M. Lund P. Weisbjerg M. R. (2018). Feed intake and milk production in dairy cows fed different grass and legume species: a meta-analysis. *Animal* 12 66–75. 10.1017/s1751731117001215 28560944

[B36] Kalla C. Nentwich H. Schlotter M. Mertens D. Wildenberger K. Döhner H. (2005). Translocation t (X; 11)(q13; q23) in B-cell chronic lymphocytic leukemia disrupts two novel genes. *Genes Chromosomes Cancer* 42 128–143. 15543602 10.1002/gcc.20131

[B37] Larson B. L. Smith V. R. (1974). *Lactation. A Comprehensive Treatise. Biosynthesis and Secretion of Milk/Diseases*, Vol. II. Cambridge, MA: Academic Press.

[B38] Lee N. K. Kim M. K. Choi J. H. Kim E. B. Lee H. G. Kang S. K. (2010). Identification of a peptide sequence targeting mammary vasculature via RPLP0 during lactation. *Peptides* 31 2247–2254. 10.1016/j.peptides.2010.09.008 20863866

[B39] Li Q. (2014). Transforming growth factor β signaling in uterine development and function. *J. Anim. Sci. Biotechnol.* 5:52. 10.1186/2049-1891-5-52 25478164 PMC4255921

[B40] Liu L. Zhou Z. Huang S. Guo Y. Fan Y. Zhang J. (2013). Zc3h12c inhibits vascular inflammation by repressing NF-κB activation and pro-inflammatory gene expression in endothelial cells. *Biochem. J.* 451 55–60. 10.1042/bj20130019 23360436 PMC3922049

[B41] Masuda Y. (2019). *Introduction to BLUPF90 suite programs: Standard Edition*. Available online at: http://nce.ads.uga.edu/wiki/doku.php?id=documentation

[B42] Matsumoto H. Daikoku T. Wang H. Sato E. Dey S. K. (2004). Differential expression of Ezrin/Radixin/Moesin (ERM) and ERM-associated adhesion molecules in the blastocyst and uterus suggests their functions during Implantation1. *Biol. Reprod.* 70 729–736. 10.1095/biolreprod.103.022764 14613898

[B43] Michalczyk A. Ackland M. L. Brown R. W. Collins J. P. (2001). Lactation affects expression of intermediate filaments in human breast epithelium. *Differentiation* 67 41–49. 10.1046/j.1432-0436.2001.067001041.x 11270122

[B44] Mohammadi K. Nassiri M. T. B. Rahmatnejad E. Sheikh M. Fayazi J. Manesh A. K. (2013). Phenotypic and genetic parameter estimates for reproductive traits in Zandi sheep. *Trop. Anim. Health Prod.* 45 671–677. 10.1007/s11250-012-0276-0 23086601

[B45] Mokhtari M. S. Rashidi A. Esmailizadeh A. K. (2010). Estimates of phenotypic and genetic parameters for reproductive traits in Kermani sheep. *Small Rumin. Res.* 88 27–31. 10.1016/j.smallrumres.2009.11.004

[B46] Moradband F. Rahimi G. Gholizadeh M. (2011). Association of polymorphisms in fecundity genes of GDF9, BMP15 and BMP15-1B with litter size in Iranian Baluchi sheep. *Asian Austr. J. Anim. Sci.* 24 1179–1183. 10.5713/ajas.2011.10453

[B47] Morgan J. E. Fogarty N. M. Nielsen S. Gilmour A. R. (2007). The relationship of lamb growth from birth to weaning and the milk production of their primiparous crossbred dams. *Aust. J. Exp. Agric.* 47 899–904. 10.1071/ea06290

[B48] Morrison B. Cutler M. L. (2010). The contribution of adhesion signaling to lactogenesis. *J. Cell. Commun. Signal.* 4 131–139. 10.1007/s12079-010-0099-6 21063503 PMC2948120

[B49] Munoz-Suano A. Hamilton A. B. Betz A. G. (2011). Gimme shelter: the immune system during pregnancy. *Immunol. Rev.* 241 20–38. 10.1111/j.1600-065X.2011.01002.x 21488887

[B50] Neupane M. Kiser J. N. Bovine Respiratory Disease Complex Coordinated Agricultural Project Research Team Neibergs H. L. (2018). Gene set enrichment analysis of SNP data in dairy and beef cattle with bovine respiratory disease. *Anim. Genet.* 49 527–538. 10.1111/age.12718 30229962

[B51] Ostankova Y. V. Klimovskaya Y. S. Gorskaya O. A. Kolobov A. V. Kvetnoi I. M. Selkov S. A. (2011). Expression of thrombospondin-1 gene mRNA and protein in the placenta in gestosis. *Bull. Exp. Biol. Med.* 151 215–218. 10.1007/s10517-011-1292-1 22238753

[B52] Palombo V. Milanesi M. Sgorlon S. Capomaccio S. Mele M. Nicolazzi E. (2018). Genome-wide association study of milk fatty acid composition in Italian Simmental and Italian Holstein cows using single nucleotide polymorphism arrays. *J. Dairy Sci.* 101 11004–11019. 10.3168/jds.2018-14413 30243637

[B53] Paten A. M. (2014). *Maternal Nutritional Programming in the Sheep: Effects on Post-Natal Growth, Mammogenesis and Lactation in Adult-Ewe Offspring: A Thesis Submitted for the Degree.* Ph.D. thesis in Animal Science. Palmerston North: Massey University.

[B54] Peuhu E. Virtakoivu R. Mai A. Wärri A. Ivaska J. (2017). Epithelial vimentin plays a functional role in mammary gland development. *Development* 144 4103–4113. 28947532 10.1242/dev.154229

[B55] R Core Team (2021). *R: A language and environment for statistical computing*. Vienna, Austria: R Foundation for Statistical Computing. Available online at: https://www.R-project.org/

[B56] Rashidi A. Mokhtari M. S. Esmailizadeh A. K. Fozi M. A. (2011). Genetic analysis of ewe productivity traits in Moghani sheep. *Small Rumin. Res.* 96 11–15. 10.1016/j.smallrumres.2010.11.001

[B57] Raven L.-A. Cocks B. G. Pryce J. E. Cottrell J. J. Hayes B. J. (2013). Genes of the RNASE5 pathway contain SNP associated with milk production traits in dairy cattle. *Genet. Sel. Evol.* 45:25. 10.1186/1297-9686-45-25 PMC3733968 23865486

[B58] Rodriguez A. Tripurani S. K. Burton J. C. Clementi C. Larina I. Pangas S. A. (2016). SMAD signaling is required for structural integrity of the female reproductive tract and uterine function during early pregnancy in mice. *Biol. Reprod.* 95 41–44. 27335065 10.1095/biolreprod.116.139477 PMC5029477

[B59] Rönnegård L. McFarlane S. E. Husby A. Kawakami T. Ellegren H. Qvarnström A. (2016). Increasing the power of genome wide association studies in natural populations using repeated measures–evaluation and implementation. *Methods Ecol. Evol.* 7 792–799. 10.1111/2041-210x.12535 27478587 PMC4950150

[B60] Rosati A. Mousa E. Van Vleck L. D. Young L. D. (2002). Genetic parameters of reproductive traits in sheep. *Small Rumin. Res.* 43 65–74. 10.1016/s0921-4488(01)00256-5

[B61] Rutten M. J. M. Bouwman A. C. Sprong R. C. van Arendonk J. A. M. Visker M. H. P. W. (2013). Genetic variation in vitamin B-12 content of bovine milk and its association with SNP along the bovine genome. *PLoS One* 8:e62382. 10.1371/journal.pone.0062382 23626813 PMC3633873

[B62] Salleh M. S. Mazzoni G. Höglund J. K. Olijhoek D. W. Lund P. Løvendahl P. (2017). RNA-Seq transcriptomics and pathway analyses reveal potential regulatory genes and molecular mechanisms in high-and low-residual feed intake in Nordic dairy cattle. *BMC Genomics* 18:258. 10.1186/s12864-017-3622-9 28340555 PMC5366136

[B63] Shamir E. R. Ewald A. J. (2015). “Adhesion in mammary development: novel roles for E-cadherin in individual and collective cell migration,” in *Current Topics in Developmental Biology*, ed. Yap A. S. (Amsterdam: Elsevier), 353–382. 10.1016/bs.ctdb.2014.12.001 PMC4696070 25733146

[B64] Sheftel A. D. Stehling O. Pierik A. J. Elsässer H.-P. Mühlenhoff U. Webert H. (2010). Humans possess two mitochondrial ferredoxins, Fdx1 and Fdx2, with distinct roles in steroidogenesis, heme, and Fe/S cluster biosynthesis. *Proc. Natl. Acad. Sci. U.S.A.* 107 11775–11780. 10.1073/pnas.1004250107 20547883 PMC2900682

[B65] Snowder G. D. Fogarty N. M. (2009). Composite trait selection to improve reproduction and ewe productivity: a review. *Anim. Prod. Sci.* 49 9–16. 10.1071/ea08184

[B66] Strid H. Powell T. L. (2000). ATP-dependent Ca 2+ transport is up-regulated during third trimester in human syncytiotrophoblast basal membranes. *Pediatr. Res.* 48:58. 10.1203/00006450-200007000-00012 10879801

[B67] Tahmoorespur M. Sheikhloo M. (2011). Pedigree analysis of the closed nucleus of Iranian Baluchi sheep. *Small Rumin. Res.* 99 1–6. 10.1016/j.smallrumres.2011.01.017

[B68] Thompson W. D. Tyrrell J. Borges M.-C. Beaumont R. N. Knight B. A. Wood A. R. (2019). Association of maternal circulating 25 (OH) D and calcium with birth weight: a mendelian randomisation analysis. *PLoS Med.* 16:e1002828. 10.1371/journal.pmed.1002828 31211782 PMC6581250

[B69] Tiezzi F. Parker-Gaddis K. L. Cole J. B. Clay J. S. Maltecca C. (2015). A genome-wide association study for clinical mastitis in first parity US Holstein cows using single-step approach and genomic matrix re-weighting procedure. *PLoS One* 10:e0114919. 10.1371/journal.pone.0114919 25658712 PMC4319771

[B70] To B. Andrechek E. R. (2018). Transcription factor compensation during mammary gland development in E2F knockout mice. *PLoS One* 13:e0194937. 10.1371/journal.pone.0194937 29617434 PMC5884531

[B71] Tra T. Gong L. Kao L.-P. Li X.-L. Grandela C. Devenish R. J. (2011). Autophagy in human embryonic stem cells. *PLoS One* 6:e27485. 10.1371/journal.pone.0027485 22110659 PMC3215747

[B72] Vatankhah M. Talebi M. A. Edriss M. A. (2008). Estimation of genetic parameters for reproductive traits in Lori-Bakhtiari sheep. *Small Rumin. Res.* 74 216–220. 10.1016/j.smallrumres.2007.02.008

[B73] Wang C. T. Dickerson G. E. (1991). Simulation of life-cycle efficiency of lamb and wool production for genetic levels of component traits and alternative management options. *J. Anim. Sci.* 69 4324–4337. 10.2527/1991.69114324x 1752808

[B74] Wang L. Jia P. Wolfinger R. D. Chen X. Zhao Z. (2011). Gene set analysis of genome-wide association studies: methodological issues and perspectives. *Genomics* 98 1–8. 10.1016/j.ygeno.2011.04.006 21565265 PMC3852939

[B75] Wattegedera S. Rocchi M. Sales J. Howard C. J. Hope J. C. Entrican G. (2008). Antigen-specific peripheral immune responses are unaltered during normal pregnancy in sheep. *J. Reprod. Immunol* 77 171–178. 10.1016/j.jri.2007.07.003 17826845

[B76] Wickham H. (2016). *ggplot2: Elegant Graphics for Data Analysis.* Cham: Springer.

[B77] Yavarifard R. Ghavi Hossein-Zadeh N. Shadparvar A. A. (2015). Estimation of genetic parameters for reproductive traits in Mehraban sheep. *Czech J. Anim. Sci* 60 281–288. 10.17221/8242-CJAS

[B78] Young M. D. Wakefield M. J. Smyth G. K. Oshlack A. (2010). Gene ontology analysis for RNA-seq: accounting for selection bias. *Genome Biol.* 11:R14. 10.1186/gb-2010-11-2-r14 PMC2872874 20132535

[B79] Zhang J. Zan L. Fang P. Zhang F. Shen G. Tian W. (2008). Genetic variation of PRLR gene and association with milk performance traits in dairy cattle. *Can. J. Anim. Sci.* 88 33–39. 10.4141/cjas07052

[B80] Zhang L. Zhao Z. Mi H. Liu B. Wang B. Yang L. (2019). Modulation of helper T cytokines in thymus during early pregnancy in ewes. *Animals* 9:245. 10.3390/ani9050245 31100843 PMC6563054

[B81] Zhao W.-X. Lin J.-H. (2012). Notch signaling pathway and human placenta. *Int. J. Med. Sci.* 9:447. 10.7150/ijms.4593 22859905 PMC3410364

